# Respiratory Syncytial Virus Associated Hepatitis in Pregnancy

**DOI:** 10.7759/cureus.31657

**Published:** 2022-11-18

**Authors:** Alexander Malik, John Abdelnour, Muhammad N Yousaf, Sami Samiullah, Veysel Tahan

**Affiliations:** 1 Internal Medicine, Summa Health System, Akron, USA; 2 Internal Medicine, Northeast Ohio Medical University (NEOMED), Akron, USA; 3 Internal Medicine, Metro Health, Cleveland, USA; 4 Gastroenterology and Hepatology, University of Missouri, Columbia, USA; 5 Gastroenterology and Hepatology, Summa Health System, Akron, USA; 6 Gastroenterology and Hepatology, Northeast Ohio Medical University (NEOMED), Akron, USA; 7 Internal Medicine/Gastroenterology and Hepatology, University of Missouri, Columbia, USA

**Keywords:** infectious diseases, hepatology, immunosuppression, respiratory syncytial virus (rsv), pregnancy, acute viral hepatitis

## Abstract

Respiratory syncytial virus (RSV) predominantly affects children and typically manifests as an upper respiratory tract infection. Primary RSV infection in immunosuppressed adults may increase risks of disseminated infection manifesting as RSV hepatitis. A 29-year-old pregnant woman of 10 weeks gestation presented with mild right upper quadrant abdominal pain, intractable nausea, and vomiting, requiring hospitalization. Due to initial lab work showing significantly elevated liver transaminases, she underwent a thorough workup to evaluate for causes of hepatitis. Common viral and autoimmune etiologies of hepatitis were excluded with appropriate serologies. A respiratory viral molecular panel (RVP) was obtained to evaluate for SARS-CoV-2/coronavirus disease 2019 (COVID-19) infection, despite lack of typical respiratory symptoms. No structural pathologies were detected on abdominal imaging with ultrasound and magnetic resonance imaging. No other etiologies for the patient’s hepatitis were detected other than RSV infection detected on RVP. The patient’s care required close coordination between multiple different subspecialties. Her condition improved due to the early detection of RSV infection and prompt initiation of supportive care. This case highlights the need for providers to consider obtaining an RVP early in workup of hepatitis to evaluate for RSV infection, even when patients have minimal respiratory symptoms. A high index of suspicion is required for early identification of RSV hepatitis as timely supportive care may prevent progression to acute liver failure.

## Introduction

Respiratory syncytial virus (RSV) predominantly affects children and typically manifests as an upper respiratory tract infection. Primary RSV infection in immunosuppressed adults may increase the risk of disseminated infection manifesting as RSV hepatitis. RSV hepatitis may present with fever, abdominal pain, nausea, vomiting, jaundice, coagulopathy, and elevation of transaminases. Reports of RSV hepatitis in the setting of pregnancy have not been well described in previous literature. Healthcare providers must have a high index of suspicion for RSV hepatitis in the setting of pregnancy to initiate early supportive care, which can prevent the progression to acute liver failure.

## Case presentation

**Figure 1:**
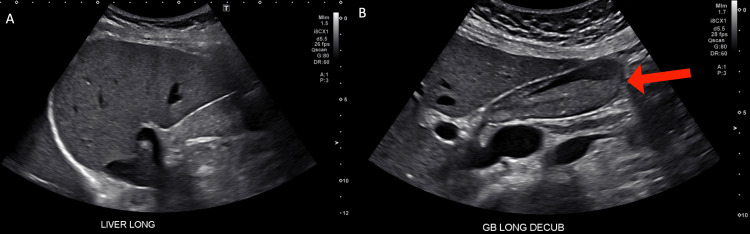
Normal abdominal ultrasound of the patient. The abdominal ultrasound images show (A) the long axis of the liver with no significant pathologies and (B) the long axis of the gallbladder (red arrow) with cholelithiasis without evidence of cholecystitis.

A 29-year-old pregnant woman of 10 weeks gestation, history of anemia, and vitamin B12 deficiency was admitted to the hospital for fatigue, mild right upper quadrant (RUQ) abdominal pain, intractable nausea, vomiting, and inability to tolerate oral intake during the winter season. She denied respiratory symptoms at the time of presentation. She was taking prenatal multivitamins and she denied taking any other medications or supplements. At presentation, her vital signs were normal. Clinical examination was significant for mild RUQ abdominal pain; there was no scleral icterus, jaundice, rashes, or epigastric pain; the uterine fundus was palpable just above the pubic bone. Initial laboratory workup revealed elevated liver enzymes with aspartate aminotransferase (AST) 497 U/L and alanine aminotransferase (ALT) 712 U/L; total bilirubin, alkaline phosphatase (ALP), and albumin were normal (Table 1). Abdominal ultrasound (US) demonstrated cholelithiasis without evidence of cholecystitis or common bile duct dilation (Figures 1A, 1B).

**Table 1:** Laboratory testing results of emesis etiology investigation. H: high; N: normal; L: low; PCR: polymerase chain reaction

Laboratory test		Reference range	Results
Liver function tests	Alanine aminotransferase (ALT)	0-34 U/L	990 (H)
	Aspartate aminotransferase (AST)	15-46 U/L	750 (H)
	Alkaline phosphatase (ALP)	38-126 U/L	89 (N)
	Total bilirubin	0.2-1.3 mg/dL	1.4 (H)
	Total protein	6.3-8.2 g/dL	6.4 (N)
	Albumin	3.5-5.0 g/dL	3.6 (N)
Coagulation studies	Prothrombin time	9.0-12.0	10.9 (N)
	International normalized ration	0.9-1.1	1.0 (N)
Viral serologies	Hepatitis A, IgM	Non-reactive	Non-reactive
	Hepatitis B, core IgM	Non-reactive	Non-reactive
	Hepatitis B, surface antigen	Non-reactive	Non-reactive
	Hepatitis C antibody	Non-reactive	Non-reactive
	Hepatitis E Antibody	Non-reactive	Non-reactive
	Human immunodeficiency virus 1 and 2 antibody/antigen	Non-reactive	Non-reactive
	Herpes simplex virus 1 and 2 IgM	≤0.89	0.96 (intermediate)
	Cytomegalovirus quantitative PCR	Non-reactive	Not detected
	Epstein-Barr virus, IgM	Not detected	Not detected
	Influenza A, antigen	Not detected	Not detected
	Influenza B, antigen	Not detected	Not detected
	Respiratory syncytial virus	Not detected	Detected
Autoimmune liver disease panel	Liver-kidney microsome-1 antibody IgG (anti-LKM)	0.0-24.9 U	0.8 (N)
	Antinuclear antibody (ANA) titer	<1:80	<1:80 (N)
	Anti-smooth muscle antibody (ASMA)	0-19 Units	6 (N)
	Antimitochondrial antibody (AMA)	0.0-24.9 Units	2.4 (N)
Miscellaneous	Rapid plasma regain (RPR)	Negative	Negative
	Total creatinine kinase (CK)	30-170 U/L	<20 (L)
	*Heliobacter pylori* antigen	Negative	Negative

**Figure 2:**
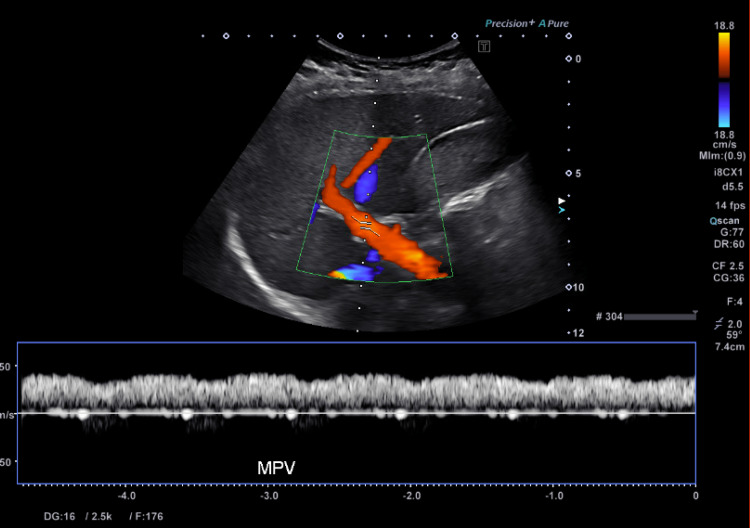
Normal abdominal ultrasound with Doppler. The image shows normal blood flow through the main portal vein. MPV: main portal vein

**Figure 3:**
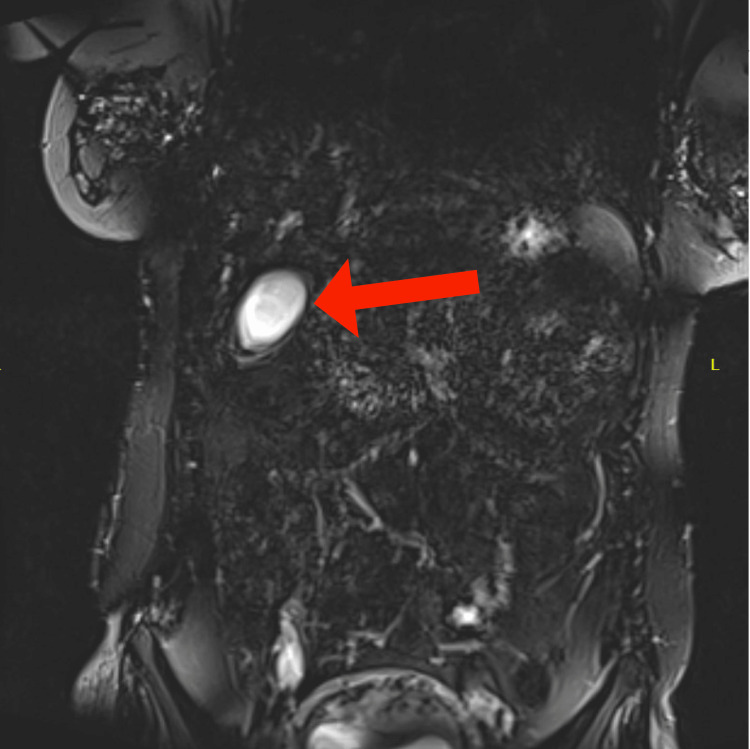
Normal abdominal MRCP of the patient. Coronal T2-weighted image from MRCP of the abdomen with normal gallbladder appearance (red arrow). MRCP: magnetic resonance cholangiopancreatography

Subsequent workup including an acute hepatitis panel, HIV, cytomegalovirus (CMV), Epstein-Barr virus (EBV) serologies, and stool *Heliobacter pylori* testing was negative. Autoimmune workup including antinuclear antibody (ANA) titer, anti-smooth muscle antibody (ASMA), antimitochondrial antibody (AMA), and liver-kidney microsome-1 antibody IgG (anti-LKM) was negative. Herpes simplex virus (HSV) IgM serology was indeterminant. Lipase was within normal limits. A respiratory viral molecular panel (RVP) by polymerase chain reaction was positive for RSV. Abdominal US with Doppler showed normal hepatic and portal vessel blood flow (Figure 2) and magnetic resonance cholangiopancreatography (MRCP) was negative for choledocholithiasis (Figure 3). There were no findings suggestive of pancreatic inflammation on abdominal US or MRCP.

While admitted to the hospital, she was treated supportively with intravenous fluids, antiemetics, and close fetal monitoring. She remained afebrile and continued to deny respiratory symptoms throughout the hospital course and had no requirements for supplemental oxygen. Her liver enzymes peaked on admission day four with AST 863 U/L, ALT 1214 U/L, and total bilirubin 1.1 mg/dL. By admission day five, her symptoms improved, and she was discharged. At five weeks follow-up, AST and ALT improved to 62 U/L and 82 U/L, respectively. An elective laparoscopic cholecystectomy was deferred until after delivery due to symptom resolution and the absence of acute cholecystitis.

## Discussion

While RSV is the most common reason for admission to the pediatric intensive care unit, disseminated RSV is a rare manifestation in adults [1-3]. Adult patients at risk of RSV infection requiring hospital admission include those with causes of immunosuppression (including pregnancy), elderly patients (age≥65 years), and those with chronic cardiac and pulmonary disorders [4,5]. Hepatitis in the setting of severe RSV has been well-described, primarily in the pediatric population [1]. The clinical presentation of RSV hepatitis may be atypical, creating diagnostic challenges. It is imperative to rule out other more common causes of acute hepatitis. This process entails conducting a thorough medication review, obtaining serologies for viral causes of hepatitis, and evaluating for autoimmune causes of hepatitis. During periods of high local transmission of upper respiratory infections, such as the winter season, obtaining an RVP testing for common viral and bacterial infections should be considered [5]. In addition to hepatitis A, B, C, and E serologies, HIV, HSV, CMV, and EBV serologies should be obtained. Autoimmune serologies include ANA, ASMA, AMA, and anti-LKM. If clinical suspicion is present, metabolic causes of hepatitis including Wilson’s disease and hereditary hemochromatosis should be evaluated with ceruloplasmin levels, and iron studies (serum iron, total iron binding capacity, and ferritin levels), respectively. Abdominal imaging to evaluate for structural causes of elevated liver enzymes such as primary sclerosing cholangitis and primary biliary cirrhosis can include abdominal US, computed tomography (CT), and MRI/MRCP. Unlike US and MRI, which are generally considered safe imaging modalities in the setting of pregnancy, CT should be avoided in pregnant patients. Abdominal US with Doppler to rule out abnormal hepatic and portal vascular flow should be considered. Liver biopsy is rarely required to establish the diagnosis [1].

As was demonstrated in the case of this patient, there may be similarities between the typical manifestations of RSV infection in adults compared to the typical manifestations of RSV infection in children. Unlike pediatric patients with severe RSV infections where the degree of liver enzyme elevation usually correlates directly with the severity of respiratory symptoms, the degree of liver enzyme elevation in adults with RSV infection may not correlate with the degree of respiratory symptoms [6]. This was apparent in the present case as the patient experienced minimal respiratory symptoms. However, similarly to the typical pattern of RSV infection in children where liver enzyme elevation peaks between hospital admission days two and four, this patient experienced peak liver enzyme elevation on admission day four [6].

The clinical course and management of RSV hepatitis in pregnancy pose a unique challenge and require close coordination between healthcare providers, including the hospitalist, obstetrician, and gastroenterologist/hepatologist. Fetal monitoring with ultrasound should be used continuously over the course of the treatment process. Conservative management of associated symptoms of RSV hepatitis and serial liver chemistries remains the mainstay and was successful in the management of this patient. If oral intake is limited due to nausea and vomiting, intravenous fluids should be initiated promptly on presentation to the hospital. Antiemetic medications including metoclopramide and promethazine may be offered in addition to vitamin B6 (pyridoxine). Use of antipyretics (e.g., acetaminophen) may be considered a component of supportive care depending on the institution, and antipyretics are commonly used in the treatment of severe RSV infections in the pediatric population [7]. Yet, use of acetaminophen has been postulated to be a contributing factor of increasing activity of catalytic enzymes (including AST and ALT) in the setting of RSV-associated hepatitis [7]. However, acetaminophen can be used safely in patients with liver disease and is generally preferred over the use of non-steroidal anti-inflammatory medications in both the setting of liver disease and the setting of pregnancy [8]. Importantly, antiviral agents have no proven efficacy in the management of RSV hepatitis [7]. Additionally, the humanized monoclonal antibody, palivizumab, has no role in the treatment of RSV in adults.

## Conclusions

Disseminated RSV is a rare manifestation that typically affects immunosuppressed adults, which may include pregnant women. The clinical course of disseminated RSV in adults shares some similarities compared to the course of severe RSV infection in children. A thorough evaluation to rule out common causes of elevated liver chemistries is warranted, including investigation of prescription and over-the-counter medications, supplements, and obtaining viral and autoimmune serologies. Standard abdominal imaging with US and/or MRI is helpful to rule out structural pathologies. Liver biopsy is rarely required to establish the diagnosis. RSV hepatitis is typically self-limited and can be treated with supportive care as antiviral agents have no proven efficacy. A high index of suspicion is required for early identification of RSV hepatitis as timely supportive care may prevent progression to acute liver failure.
